# Pain after subcutaneous implantable cardioverter-defibrillator implantation: A secondary analysis of the PRAETORIAN-DFT trial

**DOI:** 10.1016/j.hroo.2025.03.011

**Published:** 2025-03-31

**Authors:** Jolien A. de Veld, Lonneke Smeding, Mikhael F. El-Chami, Christelle Marquie, Peter Nordbeck, Anne-Floor B.E. Quast, Roland R. Tilz, Tom F. Brouwer, Pier D. Lambiase, Christopher J. Cassidy, Lucas V.A. Boersma, Martin C. Burke, Shari Pepplinkhuizen, Leonard A. Dijkshoorn, Anouk de Weger, Harish Manyam, Vincent Probst, Timothy R. Betts, Nick R. Bijsterveld, Pascal Defaye, Jan Elders, Gregory Golovchiner, Jonas S.S.G. de Jong, Nigel Lewis, Eloi Marijon, Claire A. Martin, Marc A. Miller, Naushad A. Shaik, Willeke van der Stuijt, Jürgen Kuschyk, Louise R.A. Olde Nordkamp, Anita Arya, Alida E. Borger van der Burg, Serge Boveda, Michael Glikson, Lukas Kaiser, Alexander H. Maass, Léon J.P.M. van Woerkens, Amir Zaidi, Arthur A.M. Wilde, Reinoud E. Knops

**Affiliations:** 1 Amsterdam UMC location University of Amsterdam, Heart Center, Department of Cardiology, Amsterdam Cardiovascular Sciences Heart Failure & Arrhythmias, Amsterdam, the Netherlands; 2 Section of Electrophysiology, Division of Cardiology, Emory University, Atlanta, Georgia; 3 Department of Cardiology, CHU de Lille, Lille, France; 4 Department of Internal Medicine I, University and University Hospital Würzburg, Würzburg, Germany; 5 Department of Rhythmology, University Heart Center Lübeck, University Hospital Schleswig-Holstein, Lübeck, Germany; 6 German Center for Cardiovascular Research, Partner Site Hamburg/Kiel/Lübeck, Lübeck, Germany; 7 Office of the Director of Clinical Electrophysiology Research and Lead for Inherited Arrhythmia Specialist Services, University College London and Barts Heart Centre, London, United Kingdom; 8 Lancashire Cardiac Centre, Blackpool Teaching Hospitals NHS Trust, Blackpool, United Kingdom; 9 Department of Cardiology, St. Antonius Hospital, Nieuwegein, the Netherlands; 10 CorVita Science Foundation, Chicago, Illinois; 11 Department of Cardiology, Erlanger Health System, University of Tennessee, Chattanooga, Tennessee; 12 Service de Cardiologie, L'Institut du Thorax, CHU de Nantes, Nantes, France; 13 Oxford Biomedical Research Centre, Oxford University Hospitals NHS Trust, Oxford, United Kingdom; 14 Department of Cardiology, Flevoziekenhuis, Almere, the Netherlands; 15 Service de Cardiologie, CHU Grenoble Alpes, Grenoble, France; 16 Department of Cardiology, Canisius Wilhelminahospital, Nijmegen, the Netherlands; 17 Department of Cardiology, Rabin Medical Center, Petah Tikva, Israel; 18 Department of Cardiology, OLVG, Amsterdam, the Netherlands; 19 Department of Cardiology, Sheffield Teaching Hospitals NHS Foundation Trust, Sheffield, United Kingdom; 20 Division of Cardiology, European Georges Pompidou Hospital, Paris, France; 21 Department of Cardiology, Royal Papworth Hospital, Cambridge, United Kingdom; 22 Helmsley Electrophysiology Center, Mount Sinai Hospital, Icahn School of Medicine at Mount Sinai, New York, New York; 23 Department of Cardiac Electrophysiology, Advent Health Orlando, Orlando, Florida; 24 First Department of Medicine, University Medical Center Mannheim, Mannheim, Germany; 25 First Department of Medicine-Cardiology, University Medical Center Mannheim, Mannheim, Germany; 26 German Center for Cardiovascular Research, Partner Site Heidelberg-Mannheim, Mannheim, Germany; 27 Division of Electrophysiology and Devices, Heart and Lung Centre, New Cross Hospital, Royal Wolverhampton NHS Trust, Wolverhampton, United Kingdom; 28 Department of Cardiology, Medisch Centrum Leeuwarden, Leeuwarden, the Netherlands; 29 Heart Rhythm Department, Clinique Pasteur, Toulouse, France; 30 Jesselson Integrated Heart Center, Shaare Zedek Medical Center, Jerusalem, Israel; 31 Department of Cardiology and Critical Care Medicine, Asklepios Klinik St. Georg, Hamburg, Germany; 32 Department of Cardiology, University Medical Center Groningen, Groningen, the Netherlands; 33 Department of Cardiology, Albert Schweitzer Ziekenhuis, Dordrecht, the Netherlands; 34 Manchester Heart Centre, Manchester Academic Health Sciences Centre, Central Manchester University Hospitals NHS Foundation Trust, Manchester, United Kingdom

**Keywords:** Subcutaneous ICD, Sex, Pain, Anesthesia, Implantation

## Abstract

**Background:**

The subcutaneous implantable cardioverter-defibrillator (S-ICD) overcomes transvenous lead-related complications. Its extrathoracic design results in a generator twice the size of transvenous ICDs.

**Objective:**

We investigated pain after S-ICD implantation and explore predictors for severe pain.

**Methods:**

The PRAETORIAN-DFT (PRospective randomized compArative trial of subcutanEous implanTable cardiOverter-defibrillatoR ImplANtation with and without DeFibrillation Testing) trial included 965 patients undergoing S-ICD implantation in 37 centers across Europe, the United States, and Israel. Pain was assessed using the numeric rating scale (NRS), ranging from no pain to unbearable pain. The NRS was measured before implantation, and 1 to 4 hours, 5 to 7 hours, 1 day, and 1 to 4 months after implantation. Two questions about implantation experience were asked at follow-up. Logistic regression analysis was used to identify predictors. Implanting physicians were asked their expectations on pain experience.

**Results:**

In the PRAETORIAN-DFT trial, 24% was female, mean age was 54 ± 14 years and 45% had ischemic cardiomyopathy. The median NRS within 1 day after implantation was 4. Pain was most frequently experienced at the pocket. There were 262 (29%) of 918 patients who reported severe pain (NRS ≥7) within 1 day after implantation. Predictors for severe pain were female sex (adjusted odds ratio [aOR] 2.23, *P* < .001), procedure duration >48 minutes (aOR 1.84, *P* < .001), and severe pain at baseline (aOR 3.97, *P* = .026). Additionally, female sex was a predictor for disappointment in pain perception during and after implantation. Physician anticipated NRS and location corresponded with reported pain, and females were expected to have more pain by 4 of 24 physicians.

**Conclusion:**

In the period surrounding S-ICD implantation, attention should be paid to analgesia and expectation management in patients with longer procedure duration, severe pre-existing pain, and especially female sex.

## Graphical abstract

**Figure:**
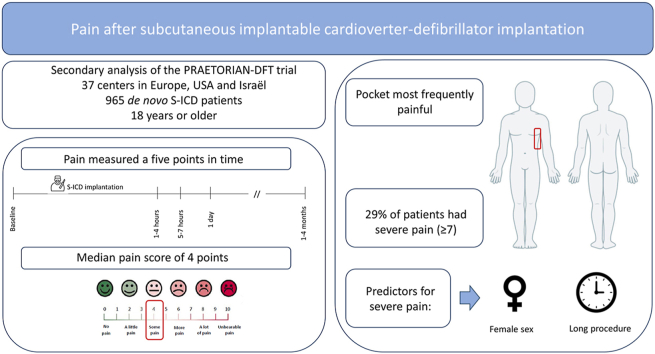

**Key Findings**

▪ Patients experience moderate pain after S-ICD implantation, with a median pain score of 4 points.
▪ The area that is most frequently reported as painful is the anterior side of the S-ICD generator pocket.
▪ Female sex, a longer procedure time, and severe pre-existing pain are predictive of severe pain after implantation.
▪ Sex-related differences in postoperative pain are not well acknowledged among implanting physicians.

## Introduction

The use of an implantable cardioverter-defibrillator (ICD) is an effective therapy to improve the survival of patients at risk for sudden cardiac death.^1^^,^^2^ The subcutaneous ICD (S-ICD) is an extrathoracic device developed to overcome the risk of serious lead-related complications associated with the transvenous ICD (TV-ICD). The S-ICD was studied extensively and shown to be a safe and successful treatment for the termination of potentially lethal ventricular arrhythmias.^3^^,^^4^

Due to the extrathoracic implant position of the S-ICD, a higher energy output is required to defibrillate the myocardium, leading to a generator that is twice the size of the TV-ICD. Besides this, the lead is tunneled on the sternum, which might be perceived as painful due to the contact with the periosteum.^5^ A randomized controlled trial between the S-ICD and TV-ICD in patients at risk of lead-related complications showed more postoperative pain in S-ICD patients, which did not persist over time.^6^^,^^7^ On the other hand, a study comparing an S-ICD cohort matched with TV-ICD patients showed no significant differences in on-demand analgesia administration.^8^ To improve postoperative pain management in S-ICD patients, it is valuable to understand which patients are prone to pain and how this could be prevented. A subanalysis of the Bridge or Continue Coumadin for Device Surgery Randomized Controlled (BRUISE-CONTROL) trial showed that patients with female sex, younger age, and lower body mass index (BMI) had significantly more postoperative pain after transvenous device implantation. It is unknown if these subgroups also experience more pain after S-ICD implantation.^9^

In the multicenter PRAETORIAN-DFT (PRospective randomized compArative trial of subcutanEous implanTable cardiOverter-defibrillatoR ImplANtation with and without DeFibrillation Testing) trial, de novo S-ICD patients were randomized between defibrillation testing (DFT) and no DFT in 37 hospitals across the United States, Europe, and Israel. Anesthesia protocols during implantation were per local routine, and data on postoperative pain were captured.^10^ In this secondary analysis, we describe postoperative pain and patient perception of S-ICD implantation and give recommendations on pain management in specific populations.

## Methods

### Patient population and study design

In the prospective, multicenter, controlled PRAETORIAN-DFT trial, 965 patients ≥18 years of age underwent de novo S-ICD implantation and were randomized to DFT or no DFT. Complete inclusion and exclusion criteria are published elsewhere.^10^ Patients were included between May 2018 and January 2023. Pain questionnaires were collected at various time points as a secondary end point. The PRAETORIAN-DFT trial was performed according to the Helsinki Declaration as revised in 2013. The protocol was approved by the local medical ethics committees of the participating institutions, and all patients provided written informed consent.

### Pain questionnaires

Patient reported pain scores and location of pain were collected at baseline, which was no more than 1 day before implantation, to correct postoperative pain scores for pre-existing pain not related to the implantation procedure. Pain was collected at 1 to 4 hours, 5 to 7 hours, 1 day, and 1 to 4 months after implantation. At 1 to 4 hours and 5 to 7 hours, questionnaires were included if completed within 10 minutes before or after the correct time window. The discharge questionnaires were only included if completed 1 day after implantation, and the follow-up questionnaires were only included if they were completed between 28 and 122 days after implantation. Pain scores were collected using the numeric rating scale (NRS), which is an 11-point rating scale with 0 representing no pain and 10 representing the worst pain possible.^11^ Besides this, patients were asked to point out the location of their pain. Two additional questions regarding the overall experience were asked 1 day and 1 to 4 months postimplantation. These were “How did you experience the pain during S-ICD implantation?” and “How did you experience the period after S-ICD implantation?” and were recorded using a 5-point Likert scale ranging from “very disappointing” to “much better than expected.”

### Anesthesia and analgesia methods

In this study, choice of anesthesia was per physician discretion and existed of monitored anesthesia care (MAC) or general anesthesia (GA). MAC was defined as local anesthesia, which involves injected agents directly to a specific area of the body, surrounding the operative site, combined with sedation and analgesia but with the preservation of spontaneous breathing and airway reflexes. GA was defined as complete, temporary loss of consciousness and protective reflexes. Ventilation is supported during GA. If used, regional anesthesia, also called a nerve block, was reported. There were no study protocols in place for pain management and nerve blocks. All procedures were performed per the hospital routine and physician’s discretion. As a result, the results in this analysis present real-world data without the interference of a study protocol for analgesia or nerve blocks.

### Pain anticipated by implanting physicians

A questionnaire was sent to all physicians who performed at least 1 S-ICD implantation in the study, to indicate their expectations of patients pain experience. This questionnaire asked the implanting physician to estimate the level of pain that they expect the patient to experience, and asked whether patient populations were treated differently due to an expected higher pain score. The full questionnaire can be found in Supplemental Appendix I.

### Statistical analysis

Descriptive statistics are presented as mean ± SD or as median (interquartile range [IQR]) for continuous variables and as numbers, proportions, and percentages for categorical variables. A Mann-Whitney *U* test was used to compare the change in pain score from baseline, between different patient groups. The experience of the S-ICD implantation between patient groups was compared using a chi-square test. Multiple linear regression was used to compare pain scores between anesthesia methods, corrected for diagnosis, age (≥65 years or <65 years), sex, prevention (primary or secondary), diabetes, atrial fibrillation, coronary artery bypass grafting, baseline NRS, nerve block, implanting center, randomization group, and number of incisions (2 or 3). Severe pain was defined as an NRS ≥7 points.^9^ To find predictors for severe pain at any time within the first day after implantation, we performed a logistic regression analysis. The variables age, sex, BMI (≥25 kg/m^2^ or <25 kg/m^2^), prevention, diabetes, procedure time >48 minutes, history of cardiac implantable electronic device (CIED), number of incisions, submuscular generator implantation, anesthesia method (MAC or GA), severe pain at baseline, nerve block, placement of the lead (standard or right sided), and randomization group were used for univariable analysis. The variables with a *P* value <.20 in the univariable analysis were used in the multivariable logistic regression analysis. Logistic regression analysis were also performed to find predictors for disappointment in the pain and period after implantation, reported at 1- to 4-month follow-up. A *P* value <.05 was considered statistically significant. Statistics were performed using IBM SPSS statistics version 28.

## Results

### Patient characteristics

Of the 965 patients included in this trial, 14 did not report an NRS within the correct time window at any time during the study. Of the remaining 951 patients, 24% were female (sex assigned at birth). The mean age was 54 ± 14 years, patients had a median BMI of 27 (IQR 24–31) kg/m^2^, and the most common underlying diagnosis for S-ICD implantation was an ischemic cardiomyopathy (45%), followed by nonischemic cardiomyopathy (35%). In this cohort, 315 (33%) patients received their ICD for secondary prevention. Table 1 gives an overview of the patient characteristics at baseline.

**Table 1:** Baseline characteristics (N = 951). Values are mean ± SD, no./total no. (%), or median (interquartile range)

Characteristic	n = 951
Age, y	54 ± 14
Female sex	229/951 (24)
Body mass index, kg/m^2^	27 (24–31)
Diagnosis	
Ischemic CMP	426/951 (45)
Nonischemic CMP	335/951 (35)
Genetic arrhythmic disease	92/951 (10)
Idiopathic VF	86/951 (9)
Congenital heart disease	12/951 (1)
Secondary prevention	315/951 (33)
Medical history	
Hypertension	411/951 (43)
Hypercholesterolemia	298/949 (31)
Diabetes mellitus	220/951 (23)
Atrial fibrillation	156/951 (16)
CABG	120/951 (13)
OHCA	321/951 (34)
Previously implanted CIED	115/951 (12)
DFT arm of study	477/951 (50)

CABG = coronary artery bypass grafting; CIED = cardiac implantable electronic device; CMP = cardiomyopathy; DFT = defibrillation test; OHCA = out-of-hospital cardiac arrest; VF = ventricular fibrillation.

### Pain scores after S-ICD implantation

**Figure 1:**
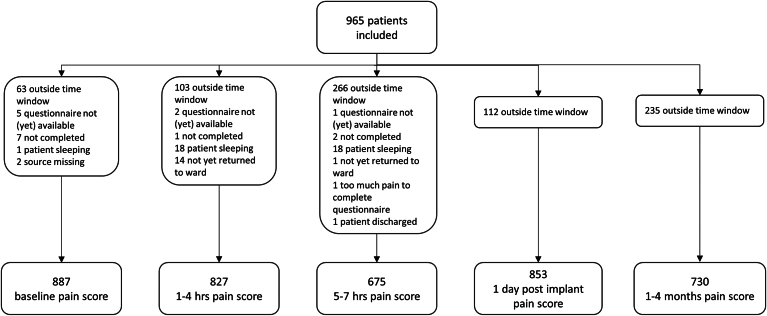
Flowchart of questionnaire completion. Number of patients who completed the questionnaire at 5 different time points. hrs = hours.

**Figure 2:**
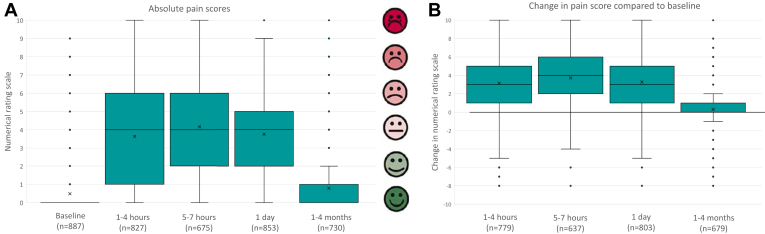
(A) Absolute pain score at different time points. The x indicates the mean score. The box indicates the interquartile range, with the inner line indicating the median. The error bars indicate the range, and outliers are presented as dots. (B) Change in pain scores from baseline. Change in pain score corrected for the baseline pain score. The zero value represents the value of pain at baseline. The x indicates the mean change score. The box indicates the interquartile range, with the inner line indicating the median. The error bars indicate the range, outliers are presented as dots. A sensitivity analysis excluding patients with a pain score other than zero at baseline showed comparable results.

**Figure 3:**
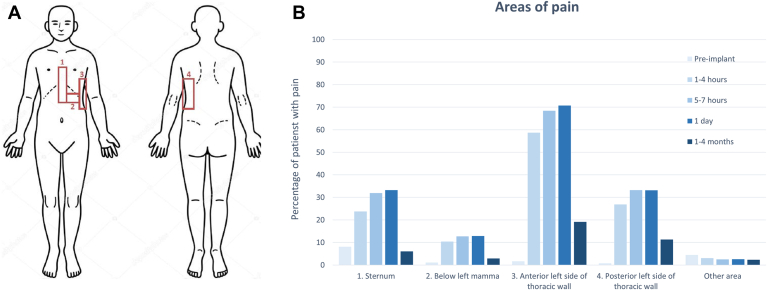
Pain areas. (A) Pain areas as presented in questionnaire. The 4 boxes indicate the areas in which patients could report pain. If patients experienced pain in other areas, this was indicated as “other.” (B) Pain areas reported at different time points. The area that was indicated as painful most frequently was the anterior side of the generator pocket, at the incision. This is most likely due to the large incision and generator, and the fact that the generator is sutured to the thoracic muscles.

A total of 918 (95%) of 965 patients completed at least 1 questionnaire within the correct time window within the first day after implantation. Questionnaires were completed by 887 (92%) of 965 patients at baseline, and by 827 (87%), 675 (70%), 853 (88%), and 730 (76%) patients at 1 to 4 hours, 5 to 7 hours, 1 day, and 1 to 4 months after implantation, respectively (Figure 1). At baseline, patients reported a median NRS of 0 (IQR 0–0) points, followed by a median NRS of 4 (IQR 1–6) at 1 to 4 hours, 4 (IQR 2–6) at 5 to 7 hours, and 4 (IQR 2–5) at 1 day after implantation. During the 1- to 4-month follow-up visit, the median NRS returned to 0 (IQR 0–1). Figures 2A and 2B give an overview of the changes in NRS at different time points, and the NRS compared with baseline. Figure 3A gives an overview of the anatomical areas in which patients could report pain. After implantation, the S-ICD pocket (area 3) was reported most frequent as painful (Figure 3B).

### Pain perception in different patient populations

Compared with men, female patients had a greater increase in pain score between baseline and every time point after implantation. This difference was significant at 5 to 7 hours and 1 day postimplantation (*P* = .037 and *P* = .001, respectively) (Supplemental Figure 1A).

Patients younger than 65 years of age had a significantly greater increase in pain score from baseline, compared with patients who were 65 years of age or older. This difference was present at any moment within 1 day after implantation, but not at the 1- to 4-month follow-up (Supplemental Figure 1B). There were no significant differences in change in pain scores between patients with a BMI <25 and ≥25 kg/m^2^ (Supplemental Figure 1C).

### Anesthesia and analgesia methods

In total, there were 463 patients who underwent their S-ICD implantation under MAC, and 488 for whom GA was used. Supplemental Table 1 gives an overview of the patient and implantation characteristics per anesthesia method.

There were no significant differences in post operative pain between patients in the MAC group and patients in the GA group. This was consistent after correction for the following confounders: diagnosis, age (≥65 years or <65 years), sex, prevention (primary or secondary), diabetes, atrial fibrillation, coronary artery bypass grafting, baseline NRS, nerve block, implanting center, randomization group, and number of incisions (2 or 3).

In total, there were 193 patients in whom nerve blocks were used during implantation. Blocks were used more frequently in the MAC group compared with the GA group (112 of 463 vs 81 of 488, *P* = .004), and the procedure time was longer in patients receiving nerve blocks (50 [IQR 40–62] minutes vs 48 [IQR 35–60] minutes, *P* = .027). Multivariable regression analysis showed no significant differences in pain scores between patients with and without nerve blocks.

### Predictors for severe pain

There were 262 (29%) of 918 patients who reported severe pain (NRS ≥7) at least once between implantation and discharge. Twelve patients reported severe pain before implantation, with 8 patients locating this pain in another area than future the S-ICD operating site. The remaining 4 patients with severe pain in the thoracic area all had a history of out-of-hospital cardiac arrest. Univariable logistic regression analysis showed that an age <65 years (odds ratio [OR] 1.48, 95% confidence interval [CI] 1.03–2.11, *P* = .032), procedure duration >48 minutes (OR 1.86 95% CI 1.39–2.48, *P* < .001), female sex (OR 2.29, 95% CI 1.67–3.14, *P* < .001), and severe pain at baseline (OR 3.83, 95% CI 1.21–12.18, *P* = .023) were univariable predictors for severe pain after implantation (Supplemental Table 2). Multivariable analysis showed the following independent predictors: female sex (OR 2.23, 95% CI 1.62–3.08, *P* < .001), severe pain at baseline (OR 3.97, 95% CI 1.18–13.36, *P* = .026), and a procedure duration > 48 minutes (OR 1.84, 95% CI 1.37–2.48, *P* < .001) (Table 2).

**Table 2:** Multivariable predictors for severe pain after implantation

Variable	Adjusted odds ratio	95% CI	*P* value
Female sex	2.23	1.62–3.08	<.001∗
Right-sided lead placement	1.50	0.70–3.23	.302
Age <65 y	1.30	0.90–1.88	.166
Procedure duration >48 min†	1.84	1.37–2.48	<.001∗
Severe pain before implantation	3.97	1.18–13.36	.026∗
Secondary prevention	0.77	0.56–1.06	.109

A sensitivity analysis excluding patients who only reported severe pain in an area not related to the subcutaneous implantable cardioverter-defibrillator showed similar results.

CI = confidence interval.

∗ These variables significantly predict severe pain after implantation.

† Procedure duration was defined as the time between the first incision and last suture.

### Patient experience

The majority of patients reported that pain during implantation was as expected or better, whereas 49 (6%) of 833 patients and 70 (10%) of 715 patients reported that pain during S-ICD implantation was disappointing, at 1 day and 1 to 4 months, respectively (Supplemental Figure 2A). The period after implantation was described as disappointing by 160 (19%) of 845 and 135 (19%) of 725 patients at 1 day and 1 to 4 months, respectively (Supplemental Figure 2B).

At the 1- to 4-month follow-up, female sex, MAC as mode of anesthesia, and severe pain within 1 day after implantation were independent predictors for a disappointing retrospective view on the pain during implantation (Supplemental Table 3). For the second question about disappointment in the period after implantation, female sex and severe pain after implantation were identified as independent predictors for disappointment (Supplemental Table 4).

### Pain anticipation by implanting physician

The questionnaire was completed by 24 physicians. The median pain score that was expected by the physicians was an NRS of 5 (IQR 3–6) points, with a median of 13% (IQR 8%–50%) of patients experiencing severe pain within the first day after implantation. In total, 9 of 24 physicians thought that >29% of patients report severe pain. The anterior side of the pocket was expected to be the location most frequently indicated as painful (14 of 24 physicians). Sex differences in pain experience were expected by 9 of 24 physicians, with 5 physicians expecting more pain in men and 4 more pain in women. Young patients were expected to experience more pain than older patients (16 of 24 physicians). This was also the case for patients with BMI <25 kg/m^2^ (11 of 24 physicians). There were no physicians who indicated that they give more or other information to specific patient groups, but 4 physicians give more or other pain medication to patients from who they expect higher pain scores.

## Discussion

This secondary analysis of the PRAETORIAN-DFT trial is the largest study to date that evaluates pain perception after S-ICD implantation. The main finding of this study is that the median pain score after implantation was moderate with an NRS of 4, with the device pocket being reported as painful most frequently. Furthermore, female sex, a procedure duration >48 minutes, and severe pain at baseline were independent predictors for severe pain within the first day after implantation.

The Avoid Transvenous Leads in Appropriate Subjects (ATLAS) study by Healey and colleagues^6^ comparing postoperative pain in S-ICD vs TV-ICD patients found a median pain score of 4.2 (IQR 4.0–4.4) points at the day of implantation, which was higher than the pain reported in TV-ICD patients and is in line with our results. Healy and colleagues measured the pain scores at 1 and 6 months after implantation, while we only collected follow-up scores at 1 to 4 months after the procedure. Compared with our 1- to 4-month follow-up scores, the pain scores measured by Healey and colleagues were higher at 1 month after implantation (1.3 [IQR 1.1–1.5]) but comparable at 6-month follow-up (0.7 [IQR 0.4–0.9]). These results indicate that patients should be informed about short-term postoperative pain after S-ICD implantation, which is higher than pain after transvenous ICD implantation, possibly due to the relatively large incision that is needed for the generator pocket and the fact that the generator is sutured to the thoracic muscles. Nevertheless, this pain diminishes over time.

### Pain perception in specific populations

Our results show that women experience a significantly larger increase in pain after S-ICD implantation than men and that female sex is an independent predictor for severe postoperative pain within the first day after implantation. This is in line with earlier data indicating that there are gender differences in general pain perception, and with the BRUISE-CONTROL trial identifying female sex as an independent predictor for postoperative pain after transvenous device implantation.^9^^,^^12^ These differences might be explained by sex-based differences in the neural circuity associated with pain.^12^ On the other hand, earlier studies have indicated that pain might be underestimated and undertreated in women in general.^13^^,^^14^ Besides this, an earlier study by van der Stuijt and colleagues^15^ showed that women report discomfort from their bra, as this interferes with the implantation location of the S-ICD. Interestingly, we identified female sex as an independent predictor for disappointment in pain during and the period after implantation. Therefore, specific attention should be paid in informing female patients about the possible pain that they may experience during and after S-ICD implantation. Besides this, implanting the generator more cranially to avoid interference with the bra could reduce discomfort in these patients.^15^ Furthermore, educating medical personnel about the sex-related differences and lowering the threshold to providing analgesia to female patients after an implantation procedure could improve the overall experience in these patients and reduce postoperative pain.

Also, patients younger than 65 years of age reported a larger increase in pain compared with baseline than older patients, but age was not shown to be an independent predictor for severe pain after implantation. In the BRUISE-CONTROL trial, <65 years of age was an independent predictor for moderate-to-severe postoperative pain. An explanation for these differing results could be that another cutoff for the predicted NRS was used and that pain in the BRUISE-CONTROL trial was only assessed at the first postoperative visit, which was a median of 12 days after implantation. With regard to other studies reflecting on age and postoperative pain, results have been conflicting, but there is a general trend toward more pain and opioid use in younger patients.^16^ This could be due to changes in opioid sensitivity that come with advancing age and younger patients resuming their daily activities earlier than older patients.^17^

When looking at patient acceptance and quality of life after S-ICD implantation, it seems that even though females and younger patients experience more pain, these patients have a positive patient acceptance of the S-ICD.^18^ Besides this, a subanalysis of the PRAETORIAN trial indicated no differences in quality of life after S-ICD and TV-ICD implantation, as well as no significant interaction between age or sex and device type. These findings point out that even though certain populations experience more pain and more disappointment than others, this does not affect overall quality of life. Therefore, whereas attention should be paid to pain and overall expectations at the counseling of these patients, it should not affect the choice of the device for said populations.^19^

### Anesthesia during S-ICD implantation

An earlier study comparing monitored anesthesia care with general anesthesia during S-ICD implantation showed that postoperative pain is similar between the 2 groups.^20^ In our cohort, pain was not significantly different between anesthesia modes, which is in line with current literature. Nevertheless, we found MAC as an independent predictor for disappointment in the pain during implantation. In our cohort, the level of sedation fluctuated within the MAC group, as there was no standardized protocol for the type and amount of medication that should be used for sedation. As a result, some patients were only lightly sedated and able to respond to external stimuli while shifting between being awake and asleep. Whereas the level of sedation was not recorded, it can be hypothesized that these patients experience the pain during the implantation as more painful, while the pain after the procedure when all patients are awake does not differ between anesthesia methods.

### Nerve blocks and postoperative pain

An earlier study by Zhang and colleagues^21^ found a lower need for adjunctive intra- and postoperative pain medication in patients with nerve blocks and showed that patients without nerve blocks had higher NRS pain scores at 24 hours after surgery. Similarly, Shariat and colleagues^22^ showed that intraoperative opioid requirements decrease when using nerve blocks vs surgical infiltration of local anesthetics, without significant differences in postoperative pain. Although we did not collect data on intra- and postoperative analgesia and therefore are unable to say anything about the used postoperative pain medication in our population, our results confirm that in a real-world data setting there is no significant difference in pain scores in patients with and without nerve blocks. This lack of difference is likely due to lower perioperative pain medication in patients with blocks, possibly in the form of opioids, which was already seen in patients with pectoral CIEDs.^23^ Considering that opioid use after CIED implantation procedures can lead to persistent opioid abuse, prescribing these should be kept at a minimum, and nerve blocks might be an efficient way to do this.^24^

### Other predictors for postoperative pain

A procedure time >48 minutes was an independent predictor for severe pain after S-ICD implantation. An earlier meta-analysis on risk factors for chronic postsurgical pain after lung and pleural surgery found an association between longer surgery duration and pain risk.^25^ A longer surgery duration may be an indicator for difficulties with the implantation and more damage to the tissue surrounding the operating site, resulting in more severe pain.

In addition, severe pain at baseline was predictive for severe pain after implantation. In these patients, pre-existing pain not related to the implantation procedure could lead to higher pain scores experienced also after the surgery. Our analysis showed that pre-existing pain was located outside the S-ICD implantation area in 8 of 12 patients, and a sensitivity analysis excluding patients who only had severe pain outside the S-ICD implantation area resulted in the same predictors for severe postoperative pain. For the patients with pre-existing pain in the thoracic region, this might have been the result of bruising of the ribs and sternum due to resuscitation, as they all had a history of out-of-hospital cardiac arrest.

### Gaps between physicians’ expectations and patients’ experience

Our results indicate that physicians’ expectations on location and median pain score after S-ICD implantation are comparable to patient-reported pain, with a median expected NRS of 5 points vs a reported NRS of 4 points. Furthermore, there was a wide range in estimated percentage of patients with severe pain. Whereas the actual percentage of patients with severe pain was higher than the median expected percentage, still 9 of 24 physicians anticipated a higher percentage than shown in our results. Importantly, whereas female sex showed to be an independent predictor for both severe pain and a disappointing implanting experience, only 4 out of 24 physicians anticipated this difference. This again underlines that sex-related differences in the S-ICD population are not well acknowledged and that more attention should be paid to counseling and perioperative pain medication in female patients.

### Limitations

First, no data were collected on intra- and postoperative analgesia or nerve block procedures. Second, there was no standardized protocol for anesthesia during the implantation procedure and the level of consciousness and amount of local anesthesia might differ within the anesthesia groups. On the other hand, this study includes real-world data on pain after S-ICD implantation from 37 centers and even more implanters in the United States and Europe, without the interference of a study protocol for pain management. This makes our results extrapolatable to the general S-ICD population. Third, we did not include data on pre-existing psychological factors, such as depression or anxiety disorders, which might influence postoperative pain perception. Fourth, study participation bias might have led to a better experience reported by the patients, although we expect that this was kept to a minimum as pain was a secondary outcome of the study and analgesia, anesthesia, and the implantation procedure apart from DFT were not protocolized. Fifth, data on complications that could have a specific effect on pain such as pocket hematoma was not collected. Last, even though this was only reported in 1 patient, not completing the questionnaire might have been due to severe pain resulting in nonresponse bias.

## Conclusion

Patients after S-ICD implantation most frequently experience pain at the generator pocket, with a median NRS of 4, which decreases to zero at 1- to 4-month follow-up. The level of pain is not dependent of the mode of anesthesia, but female sex, a longer procedure time, and pre-existing severe pain are predictive of severe pain after implantation. Attention should be paid to device positioning and analgesia surrounding S-ICD implantation, specifically in young and female patients, while also maintaining a correct generator position for effective defibrillation. These data can help physicians in their counseling and in preparing their patients for the implantation procedure.
