# Supplementary material for: Pain after subcutaneous implantable cardioverter-defibrillator implantation: A secondary analysis of the PRAETORIAN-DFT trial
Source: Heart Rhythm O2. 2025 Mar 31;6(6):799–807. doi: 10.1016/j.hroo.2025.03.011 (PMC12287946; doi:10.1016/j.hroo.2025.03.011)

**Table of contents**

[Supplemental table 1 Baseline and procedure characteristics per anesthesia method 2](#_Toc182579435)

[Supplemental table 2 Univariable predictors for severe pain in the first day after implantation 3](#_Toc182579436)

[Supplemental table 3a Univariable predictors for disappointment in the pain during implantation at 1-4 months follow-up 4](#_Toc182579437)

[Supplemental table 3b Multivariable predictors for disappointment in the pain during implantation at 1-4 months follow-up 5](#_Toc182579438)

[Supplemental table 4a Univariable predictors for disappointment in the period after implantation at 1-4 months follow-up 6](#_Toc182579439)

[Supplemental table 4b Multivariable predictors for disappointment in the period after implantation at 1-4 months follow-up 7](#_Toc182579440)

[Supplemental figure 1 Mean change scores in specific populations 8](#_Toc182579441)

[Supplemental figure 2 Patient experience of S-ICD implantation 10](#_Toc182579442)

[Appendix 1 Questionnaire to implanting physicians 11](#_Toc182579443)

# Supplemental table 1 Baseline and procedure characteristics per anesthesia method

|  | MAC (N=463) | GA (N=488) | P-value |
| --- | --- | --- | --- |
| Mean age (SD) - yrs | 55 (14) | 52 (14) | <0.001 |
| Females – no./total no. | 105/463 | 124/488 | 0.325 |
| Median body-mass index (IQR) – kg/m^2^ | 27 (24-30) | 27 (24-31) | 0.696 |
| Diagnosis – no./total no. |  |  | <0.001 |
| Ischemic CMP | 223/463 | 203/488 |  |
| Non-ischemic CMP | 136/463 | 199/488 |  |
| Genetic arrhythmic disease | 42/463 | 50/488 |  |
| Idiopathic VF | 57/463 | 29/488 |  |
| Congenital heart disease | 5/463 | 7/488 |  |
| Secondary prevention – no./total no. | 179/463 | 136/488 | <0.001 |
| Medical history – no./total no. |  |  |  |
| Hypertension | 189/463 | 222/488 | 0.146 |
| Hypercholesterolemia | 136/463 | 162/486 | 0.189 |
| Diabetes mellitus | 95/463 | 125/488 | 0.062 |
| Atrial fibrillation | 92/463 | 64/488 | 0.005 |
| CABG | 70/463 | 50/488 | 0.024 |
| OHCA | 184/463 | 137/488 | <0.001 |
| DFT-arm of study - no./total no. | 226/463 | 251/488 | 0.419 |
| Use of 3-incision technique - no./total no. | 12/463 | 21/488 | 0.149 |
| Right sided lead - no./total no. | 10/463 | 21/487 | 0.062 |
| Submuscular generator - no./total no. | 40/463 | 31/488 | 0.180 |

*SD=standard deviation; IQR=interquartile range; CMP=cardiomyopathy; VF=ventricular fibrillation; CABG=coronary artery bypass grafting; OHCA=out of hospital cardiac arrest; DFT=defibrillation test.*

# Supplemental table 2 Univariable predictors for severe pain in the first day after implantation

| Variable | Odds ratio | 95% CI | P-value |
| --- | --- | --- | --- |
| Randomized to DFT group | 1.07 | 0.80 - 1.42 | 0.650 |
| Age <65 years^†^ | 1.48 | 1.03 - 2.11 | **0.032** |
| BMI <25 kg/m^2^ | 1.13 | 0.84 - 1.52 | 0.419 |
| Diabetes mellitus | 0.96 | 0.69 - 1.35 | 0.827 |
| Female sex^†^ | 2.29 | 1.67 - 3.14 | **<0.001** |
| History of CIED | 1.01 | 0.66 - 1.56 | 0.959 |
| 3-incision technique | 0.85 | 0.38 - 1.92 | 0.703 |
| MAC as anesthesia | 1.20 | 0.90 - 1.60 | 0.208 |
| Nerve block | 1.18 | 0.84 - 1.67 | 0.341 |
| Right sided lead^†^ | 1.74 | 0.83 - 3.63 | 0.143 |
| Severe pain before implantation^†^ | 3.83 | 1.21 - 12.18 | **0.023** |
| Procedure duration >48 min^†^ | 1.86 | 1.39 - 2.48 | **<0.001** |
| Secondary prevention^†^ | 0.76 | 0.56 - 1.03 | 0.079 |
| Submuscular generator | 1.28 | 0.77 - 2.15 | 0.343 |

^†^These variables were included in the multivariable analysis

*DFT=defibrillation test; BMI=body-mass index; CIED=cardiac implanted electronic device; MAC=monitored anesthesia care.*

# Supplemental table 3a Univariable predictors for disappointment in the pain during implantation at 1-4 months follow-up

| Variable | Odds ratio | 95% CI | P-value |
| --- | --- | --- | --- |
| Randomized to DFT group | 1.07 | 0.65 - 1.75 | 0.792 |
| Age <65 years^†^ | 1.95 | 0.95 - 4.02 | 0.071 |
| BMI <25 kg/m^2^ | 0.95 | 0.56 - 1.60 | 0.834 |
| Diabetes mellitus | 0.71 | 0.37 - 1.35 | 0.295 |
| Female sex^†^ | 2.16 | 1.30 - 3.61 | **0.003** |
| History of CIED | 1.04 | 0.48 - 2.27 | 0.915 |
| 3-incision technique^‡^ | NA | NA | 0.998 |
| MAC as anesthesia^†^ | 1.83 | 1.10 - 3.02 | **0.019** |
| Nerve block | 1.10 | 0.59 - 2.05 | 0.756 |
| Right sided lead | 0.40 | 0.05 - 2.99 | 0.370 |
| Severe pain before implantation | 1.54 | 0.18 - 13.01 | 0.690 |
| Severe pain after implantation^†^ | 2.79 | 1.69 - 4.61 | **<0.001** |
| Procedure duration >48 min | 0.99 | 0.60 - 1.62 | 0.966 |
| Secondary prevention^†^ | 1.54 | 0.94 - 2.55 | 0.090 |

^†^These variables were included in the multivariable analysis

^‡^ There were not enough events to calculate and odds ratio for the variable 3-incision technique

*DFT=defibrillation test; BMI=body-mass index; CIED=cardiac implanted electronic device; MAC=monitored anesthesia care.*

# Supplemental table 3b Multivariable predictors for disappointment in the pain during implantation at 1-4 months follow-up

| Variable | Adjusted odds ratio | 95% CI | P-value |
| --- | --- | --- | --- |
| Female sex | 1.78 | 1.04 - 3.03 | **0.035** |
| Age <65 years | 1.80 | 0.86 - 3.78 | 0.120 |
| MAC as anesthesia | 1.94 | 1.15 - 3.27 | **0.013** |
| Severe pain after implantation | 2.71 | 1.61 - 4.56 | **<0.001** |
| Secondary prevention | 1.54 | 0.91 - 2.61 | 0.105 |

*MAC=monitored anesthesia care.*

# Supplemental table 4a Univariable predictors for disappointment in the period after implantation at 1-4 months follow-up

| Variable | Odds ratio | 95% CI | P-value |
| --- | --- | --- | --- |
| Randomized to DFT group | 1.10 | 0.76 - 1.60 | 0.621 |
| Age <65 years^†^ | 1.48 | 0.90 - 2.43 | 0.121 |
| BMI <25 kg/m^2†^ | 1.43 | 0.97 - 2.09 | 0.071 |
| Diabetes mellitus | 0.92 | 0.58 - 1.45 | 0.711 |
| Female sex^†^ | 2.22 | 1.50 - 3.30 | **<0.001** |
| History of CIED | 1.29 | 0.74 - 2.26 | 0.378 |
| 3-incision technique | 1.04 | 0.39 - 2.82 | 0.935 |
| MAC as anesthesia | 1.24 | 0.85 -1.80 | 0.262 |
| Nerve block | 1.17 | 0.73 - 1.86 | 0.519 |
| Right sided lead | 0.83 | 0.28 - 2.45 | 0.730 |
| Severe pain before implantation^†^ | 4.47 | 1.10 - 18.12 | **0.036** |
| Severe pain after implantation^†^ | 2.84 | 1.93 - 4.18 | **<0.001** |
| Procedure duration >48 min | **1.04** | 0.72 - 1.51 | 0.836 |
| Secondary prevention^†^ | **1.30** | 0.88 - 1.91 | 0.186 |

^†^These variables were included in the multivariable analysis

*DFT=defibrillation test; BMI=body-mass index; CIED=cardiac implanted electronic device; MAC=monitored anesthesia care.*

# Supplemental table 4b Multivariable predictors for disappointment in the period after implantation at 1-4 months follow-up

| Variable | Adjusted odds ratio | 95% CI | P-value |
| --- | --- | --- | --- |
| Female sex | 1.77 | 1.17 - 2.69 | **0.007** |
| Age <65 years | 1.26 | 0.75 - 2.01 | 0.379 |
| BMI <25 kg/m^2^ | 1.26 | 0.84 - 1.89 | 0.265 |
| Severe pain before implantation | 2.84 | 0.65 - 12.35 | 0.165 |
| Severe pain after implantation | 2.60 | 1.74 - 3.88 | **<0.001** |
| Secondary prevention | 1.34 | 0.89 - 2.02 | 0.157 |

*BMI=body-mass index.*

# Supplemental figure 1 Mean change scores in specific populations

1.
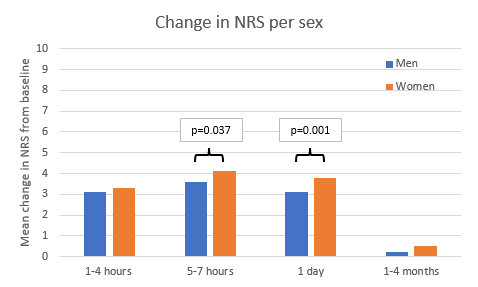
**Sex**
2.
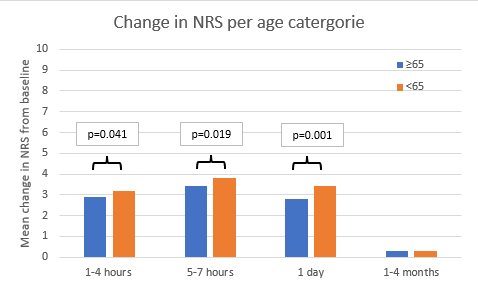
**Age**
3.
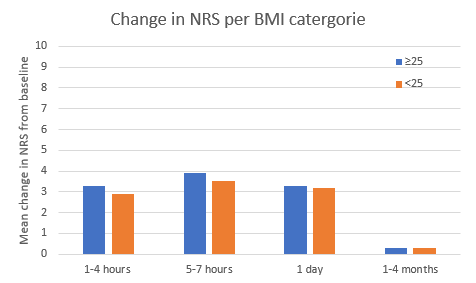
**Body-mass index**

*Only significant p-values are presented. NRS=numerical rating scale; BMI=body-mass index.*

# Supplemental figure 2 Patient experience of S-ICD implantation

1. **
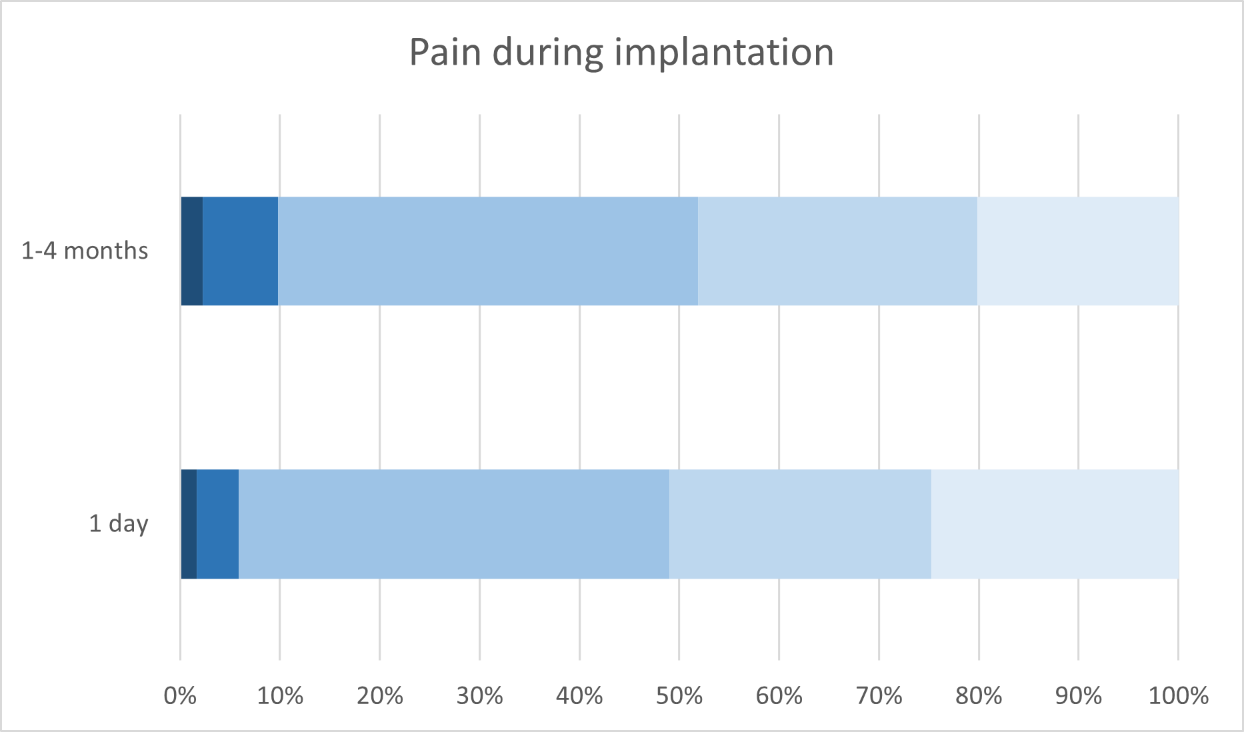
**
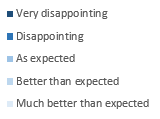
**Pain during implantation**
2. **Period after implantation**


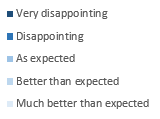
**
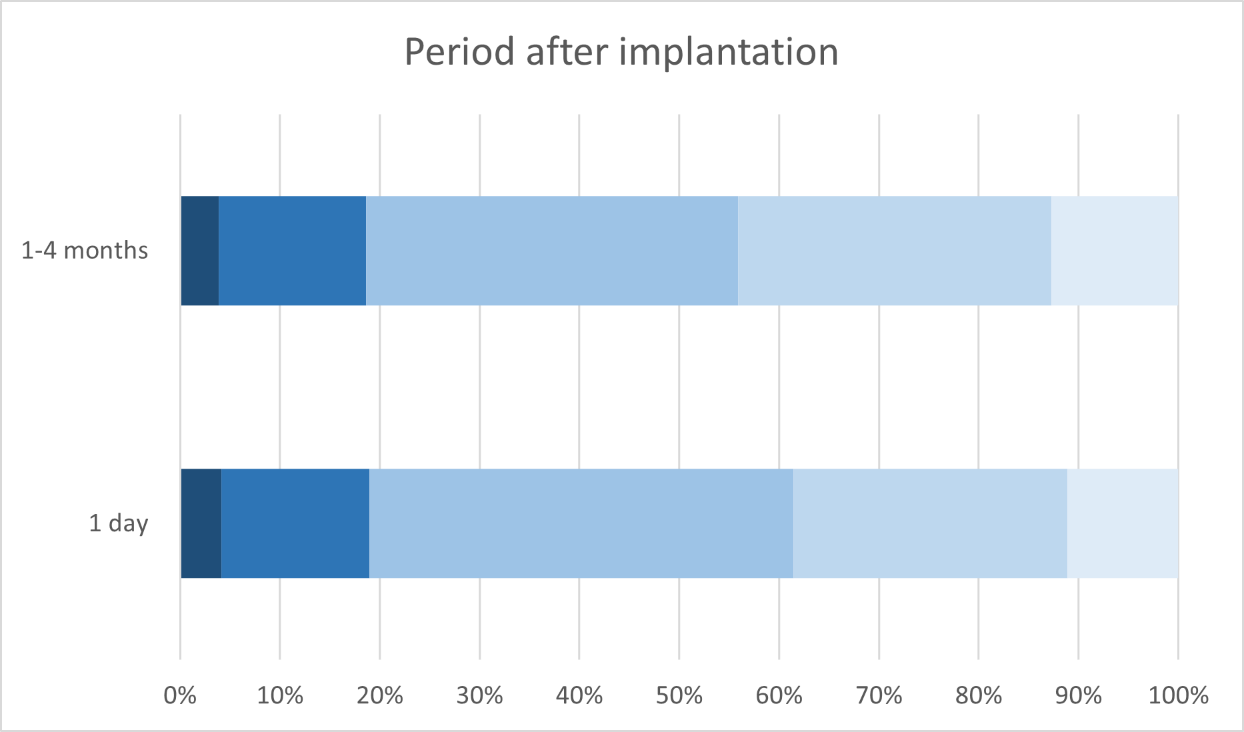
**

# Appendix 1 Questionnaire to implanting physicians


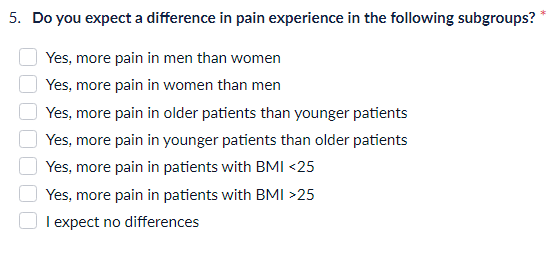

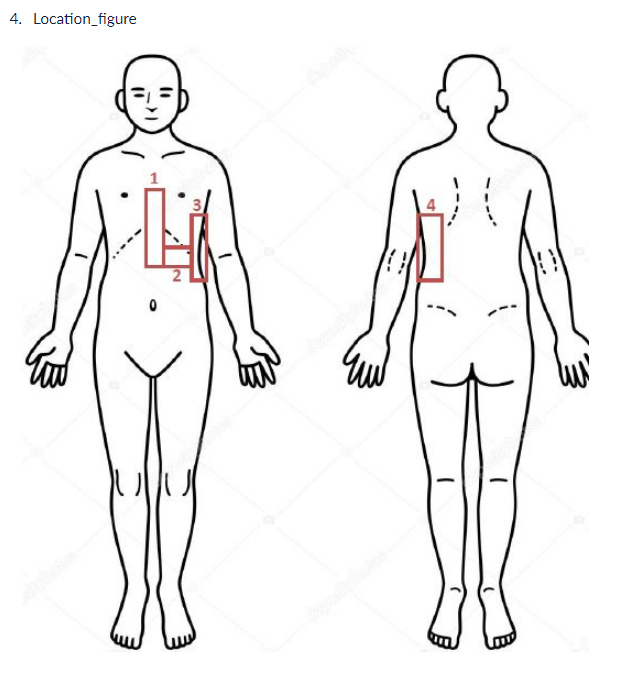

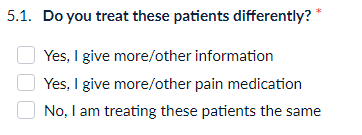

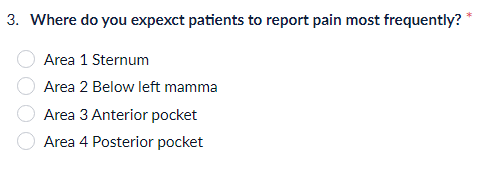

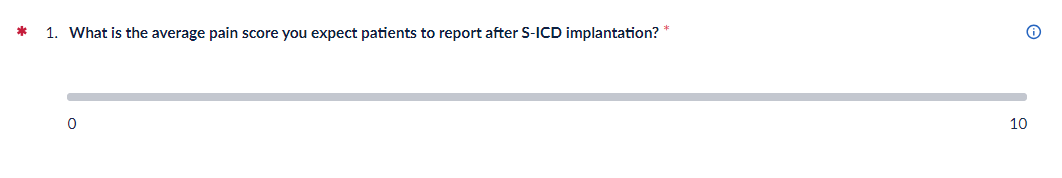

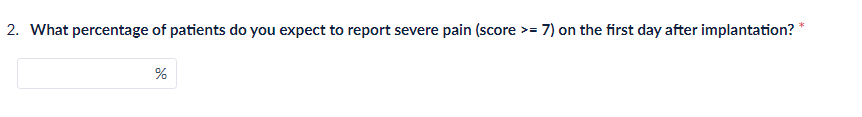

Supplement: Supplementary Material — Supplementary Data [file mmc1.docx]
